# Using Accelerometry for Evaluating Energy Consumption and Running Intensity Distribution Throughout a Marathon According to Sex

**DOI:** 10.3390/ijerph17176196

**Published:** 2020-08-26

**Authors:** Carlos Hernando, Carla Hernando, Ignacio Martinez-Navarro, Eladio Collado-Boira, Nayara Panizo, Barbara Hernando

**Affiliations:** 1 Sport Service, Jaume I University, 12071 Castellon, Spain; 2 Department of Education and Specific Didactics, Jaume I University, 12071 Castellon, Spain; 3 Department of Mathematics, Carlos III University of Madrid, 28911 Leganés, Madrid, Spain; cahernan@math.uc3m.es; 4 Department of Physical Education and Sport, University of Valencia, 46010 Valencia, Spain; ignacio.martinez-navarro@uv.es; 5 Sports Health Unit, Vithas-Nisa 9 de Octubre Hospital, 46015 Valencia, Spain; 6 Faculty of Health Sciences, Jaume I University, 12071 Castellon, Spain; colladoe@uji.es (E.C.-B.); nayapanizo@gmail.com (N.P.); 7 Department of Medicine, Jaume I University, 12071 Castellon, Spain; hernandb@uji.es

**Keywords:** accelerometry, sex, physical activity, running intensity, energy consumption, pacing, marathoners, running economy

## Abstract

The proportion of females participating in long-distance races has been increasing in the last years. Although it is well-known that there are differences in how females and males face a marathon, higher research may be done to fully understand the intrinsic and extrinsic factors affecting sex differences in endurance performance. In this work, we used triaxial accelerometer devices to monitor 74 males and 14 females, aged 30 to 45 years, who finished the Valencia Marathon in 2016. Moreover, marathon split times were provided by organizers. Several physiological traits and training habits were collected from each participant. Then, we evaluated several accelerometry- and pace-estimated parameters (pacing, average change of speed, energy consumption, oxygen uptake, running intensity distribution and running economy) in female and male amateur runners. In general, our results showed that females maintained a more stable pacing and ran at less demanding intensity throughout the marathon, limiting the decay of running pace in the last part of the race. In fact, females ran at 4.5% faster pace than males in the last kilometers. Besides, their running economy was higher than males (consumed nearly 19% less relative energy per distance) in the last section of the marathon. Our results may reflect well-known sex differences in physiology (i.e., muscle strength, fat metabolism, VO_2max_), and in running strategy approach (i.e., females run at a more conservative intensity level in the first part of the marathon compared to males). The use of accelerometer devices allows coaches and scientific community to constantly monitor a runner throughout the marathon, as well as during training sessions.

## 1. Introduction

Marathons have growth in popularity and therefore in participants worldwide at a record pace [1,2,3]. However, the increase in the number of female marathoners has been delayed, as compared to male, due to different social and behavioral causes previously pointed out by Joyner and colleagues in 2017 [4]. Although sex ratios are still far of being equivalent (i.e., 18.7% of females from the total number of participants in the Valencia Marathon 2019), female’s participation in marathon races has increased exponentially since Kathy Swizer finished the Boston Marathon in 1967.

This situation has encouraged scientific community to study female’s behavior in long-distance races and compare them with males. Studies have been focused on analyzing different factors affecting running performance such as running speed [5,6,7,8], pacing [9], physiological traits [4,10], running economy [7,11,12] and predominant type of metabolism used [13,14,15], as well as physical, biomechanical, psychological and social factors [16,17,18,19].

The assessment of physiological parameters affecting running performance has been carried out in lab-based conditions—normally by measuring the volume of expired gases (the gold standard test) [11,12,13,19]. However, lab-based conditions are far from normal race conditions. Up to now, field-based studies have been focused on estimating the energy consumption throughout a long-distance race by analyzing changes in running speed [7,10,20,21,22,23]. The use of portable measurement systems to obtain parameters for estimating energy consumption in real conditions is nowadays a reality [24,25,26,27,28,29].

In particular, the use of triaxial accelerometry has strongly emerged as a tool that allows the evaluation of a physical activity, in terms of duration, frequency and intensity, performed by an individual in free-living conditions [30,31,32,33]. Thus, using the cut-off points previously established for a specific population and/or an activity, the accelerometer output data allowed to indirectly estimate the energy cost of an activity [34,35,36,37,38].

With the aim of monitoring middle-aged recreational marathoners during a marathon using accelerometry-based devices, our research group has established the GENEActiv^®^ cut-off points, under lab-based conditions, for discriminating the six relative-intensity activity levels in female and male marathoners [39]. Once cut-off points were established, we used accelerometer output data for analyzing the running intensity distribution and energy consumption of runners during a marathon race (a free-living condition) [40]. Interestingly, accelerometer output data can also be used for inferring other useful parameters (i.e., running economy of the runner) in real conditions.

Since sex was not taken into account in our previous work, this study focused on evaluating several accelerometry-estimated parameters (energy consumption, running intensity distribution and running economy) according to the individual’s sex. The use of accelerometers allowed us to directly and constantly monitor a total of 88 recreational marathon runners (74 males and 14 females) throughout the marathon race. Here, accelerometry- and pace-based data collected from females and males were analyzed separately. In this study, we also pointed out the valuable additional information that accelerometry offers to athletes, coaches and scientific community, as compared to the evaluation of running speed.

## 2. Materials and Methods

### 2.1. Sample Set and Data Collection

From all participants of the Valencia Fundación Trinidad Alfonso EDP 2016 Marathon (20 November 2016), a total of 103 recreational marathon runners, aged 30 to 45 years, were selected to participate in this study. Eight runners did not start the race and were discarded from our study population. Finally, a total of 88 recreational marathon runners (74 males and 14 females) crossed the finish line of the Valencia Fundacion Trinidad Alfonso EDP 2016 Marathon and thus were analyzed in this study. The entire process of sampling, as well as the weather and track conditions of the race, has been previously described [41].

Details of data collection, processing and analysis have been previously described [40]. Four weeks before the marathon, participants completed a cardiopulmonary test. In this appointment, we also collected anthropometric data, demographics, medical information, training program and competition history. One hour before the marathon, all participants were weighed. During the race, participants wore a GENEActiv accelerometer (Activinsights Ltd., Kimbolton, Cambridgeshire, United Kingdom) on the non-dominant wrist as a watch. Accelerometers were adjusted to record acceleration data at a rate of 85.7 Hz, and data was summarized into Signal Vector Magnitude gravity subtracted (SVMgs) per minute. Recording and processing of acceleration data has been previously explained in detail [39]. All individuals underwent the same testing under the same experimental conditions. Raw data of this study is available in Supplementary File S1.

All individuals included in the current study were fully informed and gave their written consent to participate. All experiments were performed in accordance with international guidelines and regulations that govern human research. The research was approved by the Research Ethics Committee of the University Jaume I of Castellon and is enrolled in the ClinicalTrails.gov database (NCT03155633).

### 2.2. Data Analysis

The marathon race was divided into nine sections as previously described [40]. All analyses were performed for each of the nine race sections, as well as for the entire marathon distance.

Firstly, the physical effort distribution of each runner throughout the marathon, in terms of relative intensity levels of physical activity, was estimated using accelerometry-based devices and following the methodology previously described by our research group [39]. Different cut-off points were used to discriminate the six relative-intensity activity levels in female and male recreational marathoners (Table 1). Then, we estimated the time of each participant running at each one of the six-relative intensity levels (sedentary, light, moderate, vigorous, very vigorous and extremely vigorous). This estimation was performed for each of the nine race sections and for the whole race (Tables S1 and S2).

Next, energy consumption was calculated by using the median %VO_2max_ value of the range delimiting each intensity category in males and females (Table 1). That was applied for all intensity levels except for the sedentary category, in which the standing oxygen cost (4.5 mLO_2_·kg^−1^·min^−1^) was applied as reference value in males [42]. The reference value corresponds to the 8.1% of the maximum oxygen uptake in our males. For females, we used the 8.1% of the maximum oxygen uptake seen in our females as the VO_2_ standing (3.91 mLO_2_·kg^−1^·min^−1^). Following previous recommendations [21], we considered that one MET is equal to 3.5 mLO_2_·kg^−1^·min^−1^, and one MET is equal to one kcal·kg^−1^·h^−1^.

As the energy consumption depends on the individual’s body mass, we calculated (i) the calories consumed per kilogram of body weight per minute (kcal·kg^−1^·min^−1^), in order to obtain the physical effort intensity [20,21,43]; and (ii) the calories consumed per kilogram of body weight per kilometer (kcal·kg^−1^·km^−1^), to infer the running economy of runners [16,44]. Indeed, accelerometry-derived data was also used for estimating the %VO_2max_ maintained during the marathon by each runner, an indicator of the physical effort degree respect to the maximum value [19,42,43,45]. Following the methodology described previously, we also inferred the VO_2net_ and the energy of cost running above standing (Cr_net_) for each participant included in the study [12,46,47]. These estimations were performed by applying the corresponding reference values for females and males.

The split-times in minutes of the marathon sections were recorded for calculating the average running speed of all sections and the whole marathon distance. Then, the average change in speed (ACS) for each segment, related to the average race speed, was calculated. The average change in speed through the whole race was estimated by averaging the ACS values of all sections. The ACS is a valuable measure for assessing maintenance of running pace [9,48].

The squat jump test was performed to measure the runner’s strength before and after the race. Jumping height was estimated using the flight time of the jump, which was measured by a contact platform (Chronojump, Barcelona, Spain) [49]. Individuals were familiarized with the test’s procedure before to carry out it. Before the marathon race, all individuals performed a total of three jumps, and the best jump was recorded. After crossing the finish line, the number of attempts was conditioned by the capacity of each runner to jump (due to muscular fatigue). No more than three attempts were performed per runner, and again only the best jump was recorded.

Sex comparisons were performed by calculating the percentage of differences (gap) in all measures between males and females, as previously proposed [7,10]. Briefly, we applied the following formula: Gap = ((X_females_ − X_males_)/X_females_) × 100.

### 2.3. Statistical Analysis

Statistical analyses were done using the IBM SPSS Statistics v.26 software, and null hypothesis was rejected when the two-sided *p*-value was lower than 0.05.

The Kolgomorov–Smirnov test was used for testing data normality. Since variables were not normally distributed, all statistical analyses were performed by applying non-parametric statistical tests. The Chi-squared test was used for comparing categorical variable between males and females, while the Mann–Whitney U test was applied for comparing quantitative variables between sex groups. The meaningfulness of the outcomes was additionally estimated by inferring its effect size via the calculation of Cohen’s d, as following described [50,51]. Outcomes with values of d lower than 0.5 were considered to have an small relevance; those with d values between 0.5 and 0.8 presented moderate relevance; and those with values greater than 0.8 had large significance [52].

## 3. Results

A detailed description of individuals included in this study is summarized in Table 2. Sex differences were observed for several physiological traits, such as the body mass index (BMI; *p*-value = 0.001), the percentage of body fat (*p*-value = 2.03 × 10^−5^), the maximum oxygen uptake (VO_2max_; *p*-value = 6.59 × 10^−6^), and the maximum metabolic equivalent of task (MET; *p*-value = 6.77 × 10^−6^). Moreover, sex differences were also observed in the sessions of training performed per week (*p*-value = 0.04) and in the number of marathons completed (*p*-value = 0.03).

Using the squat jump test, we measured the level of lower body strength of each runner before and after running the marathon (Table 3). As expected, the basal squat jump height was significantly higher in males compared to females (27.34 ± 4.28 cm versus 23.84 ± 3.82 cm; *p*-value = 0.007; Cohen’s d = 0.60). No significant sex differences in the squat jump height were observed after crossing the finish line (21.88 ± 6.19 cm versus 20.53 ± 6.72 cm; *p*-value = 0.300; Cohen’s d = 0.22). Therefore, the lower body strength of females seemed to be less altered by running a marathon, one of the most challenging endurance competitions.

**Figure 1:**
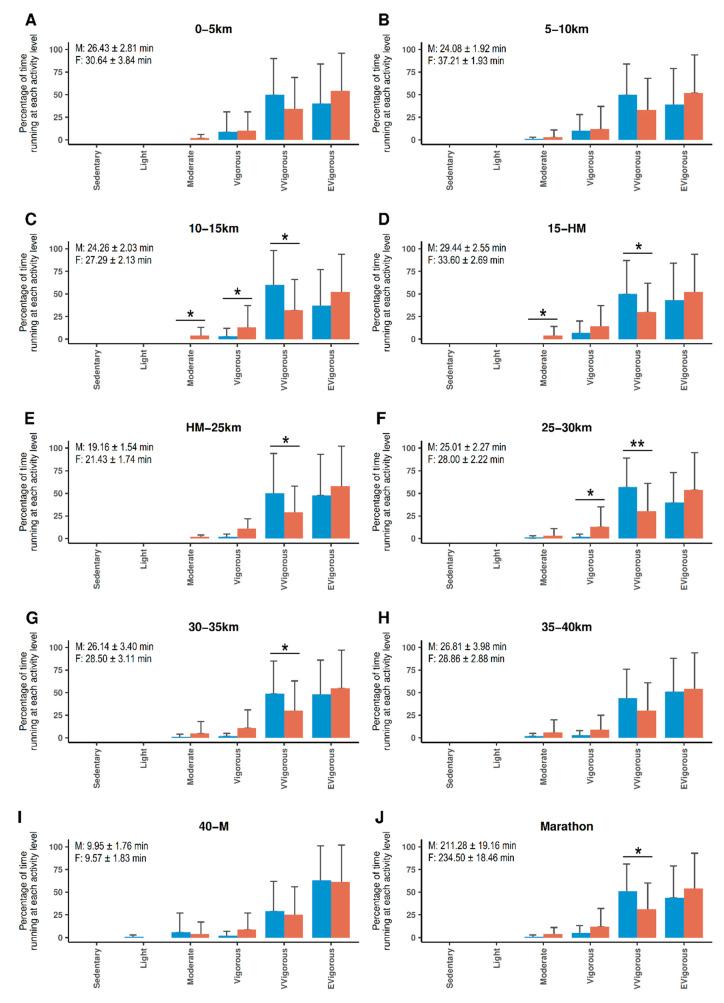
Bar plots showing the percentage of time performing at each of the six-relative intensity levels of physical activity. Running intensity distribution was analyzed (**A**–**I**) in each one of the nine race sections, and (**J**) for the whole marathon distance. Bars represent the average values for males (orange) and females (blue), and error bars represent the standard deviation of the mean. Mean time spent (±standard deviation) to cover each race sections and the whole race by males (M) and females (F) is showed in the corresponding panel. A Mann–Whitney U test was used for testing sex differences. * *p*-value < 0.05; ** *p*-value < 0.01.

Firstly, accelerometer-derived data was used to determine the running intensity distribution of female and male runners throughout the marathon. In this regard, the time consumed at each intensity level was expressed as the percentage respect to the total time needed for covering each one of the nine race sections and to the marathon time (Figure 1 and Table 3).

Time racing at extremely vigorous intensity level was similar in females and males at all race sections and in the entire marathon distance (Figure 1 and Table 3). However, males tended to spend more time running at extremely vigorous intensity than females, except for the last race section (from 40km to the finish line) (61.05 ± 40.69 % versus 62.93 ± 38.08%, respectively). Regarding the distribution of time running at the very vigorous intensity level, females seemed to spend a higher percentage of time running at this intensity level than males, with significant differences in the 10–15 km section, the 15-HM section, the HM-25 km section, the 25–30 km section, the 30–35 km section and the entire marathon distance (Figure 1 and Table 3).

Nevertheless, males were more time running at vigorous intensity than females, showing significant differences in the 10–15 km and the 25–30 km race sections (Figure 1). There were sex differences in the percentage of time running at moderate intensity in the 10–15 km and the 15-HM race sections, with males presenting higher values than females. As for the extremely vigorous level of physical intensity, females showed a higher, but not significantly, percentage of time running at moderate intensity than males in the last race section (from 40 km to the finish line) (5.50 ± 20.58% versus 4.41 ± 13.23%, respectively).

**Figure 2:**
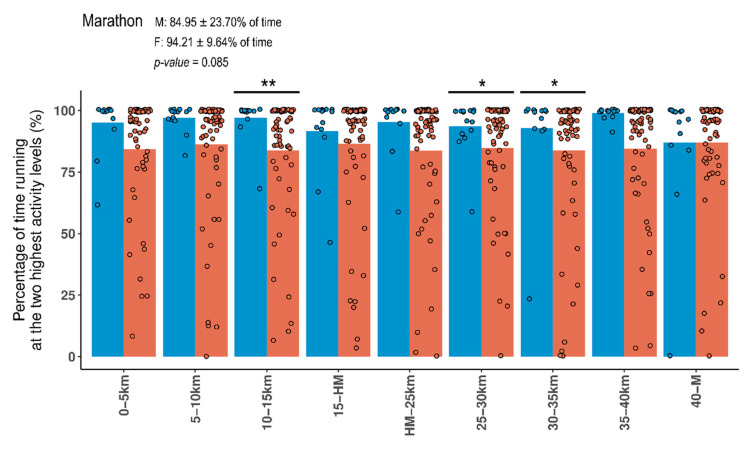
Bar plot showing the percentage of time performing at the two highest intensity levels of physical activity. Bars represent the average values for males (orange) and females (blue). Dots represent each runner included in our study. Mean percentage (±standard deviation) of time performing at these intensity levels is showed in the corresponding panel. A Mann–Whitney U test was used for testing differences between females (F) and males (M). * *p*-value < 0.05; ** *p*-value < 0.01.

As expected by the conditions of the activity, the percentage of time running at both sedentary and light intensities was minimum for both males and females. In fact, the time running at the two highest intensity levels (very vigorous and extremely vigorous) represented the 84.95 ± 23.70% of the marathon time for males and the 94.21 ± 9.64% for females (Figure 2). Therefore, running at these high intensities is crucial for achieving the marathon goal time of runners. The differences in absolute percentages denoted that females had a minimum decay rate in their running intensity, while males tended to drop from running at a very high intensity level (mainly at extremely vigorous) to a vigorous intensity level.

**Figure 3:**
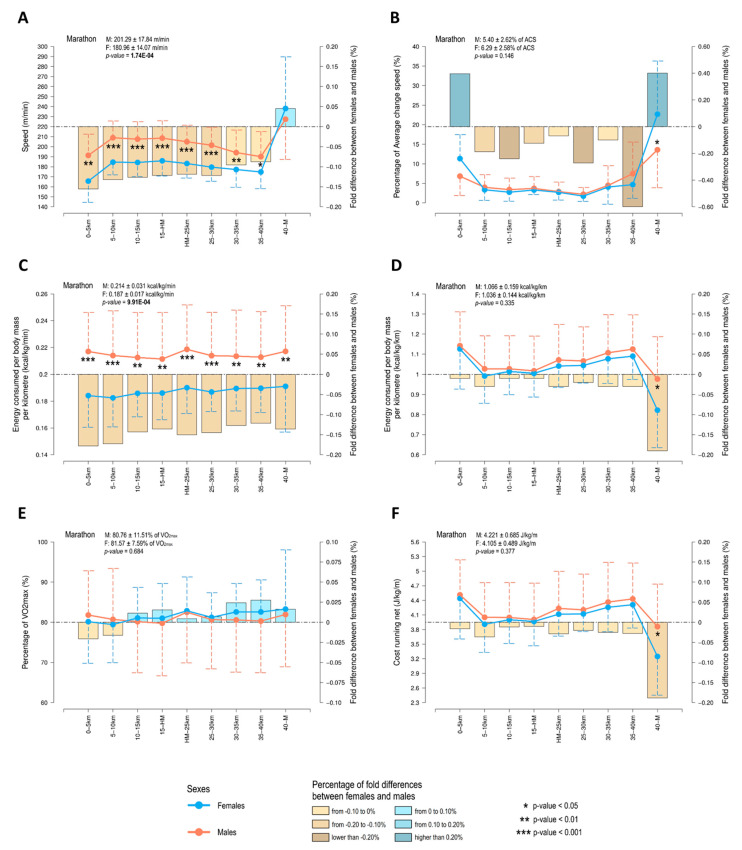
Evolution of (**A**) speed, (**B**) percentage of average change speed, (**C**) relative energy consumed per minute, (**D**) relative energy consumed per kilometer, (**E**) percentage of maximum oxygen uptake, and (**F**) cost running above the standing level. Dots represent the average values for males (orange) and females (blue), and error bars represent the standard deviation of the mean. Bars represent the percentage of sex difference (Gap). Mean values (±standard deviation) of each variable analyzed for the whole marathon distance are showed in the corresponding panel. A Mann–Whitney U test was used for testing sex differences. Bold denotes significant differences in values obtained for the whole marathon distance between females (F) and males (M).

We also compared the evolution of running speed throughout the marathon race (Figure 3A and Table 3). Overall, running speed was higher in males than in females, except for the last section of the race (from 40 km to the finish line) (238.16 ± 51.50 m·min^−1^ for females versus 227.44 ± 40.16 m·min^−1^ for males). In fact, the Gap (percentage of the relative sex difference) in running speed was decreasing during the course of the marathon. For the entire marathon distance, the running speed of females was an 11.24% slower than males (Gap = −11.24%). However, a positive Gap value (4.50%) was obtained in the last race section (from 40 km to the finish line), denoting the faster speed achieved by females compared to males in the last kilometers.

Additionally, we evaluated the evolution of ACS (Figure 3B). Results denoted higher changes of speed in females compared to males (6.29 ± 2.58 versus 5.40 ± 2.62, respectively). This observation is mainly caused by the significant increase of running speed made by females in the last section of the race. In fact, ACS values were lower in females compared to males (4.24 ± 1.75 versus 4.37 ± 2.68, respectively) when the ACS was evaluated without taking into account the last race section. That is, running pace of females was more stable than males in the first 40 km of the race and, for that reason, they were able to sprint in the last section of the marathon. Our results denoted significant sex differences in the ACS only in this last section of the race (the unique race section without significantly sex differences in the absolute running speed; Figure 3A).

Energy consumption was also compared between females and males. Males consumed 12% to 18% more energy (kilocalories normalized per body mass) per minute than females at all marathon sections (Figure 3C) and in the entire race distance (Table 3).

Accelerometry-derived data was also used to estimate the %VO_2max_ sustained throughout the race by each runner, following the methodology previously published by our research group [38]. Females and males consumed a similar %VO_2max_ at the different race sections analyzed (Figure 3E). Besides, very similar overall values were observed in males (80.76 ± 11.51% of VO_2max_) and females (81.57 ± 7.59% of VO_2max_) (Table 3). However, it is noted that females needed to maintain a slightly higher %VO_2max_ for running at a lower running intensity level in comparison to males (at very vigorous and at extremely vigorous intensity for females and males, respectively).

Running economy was measured by estimating both the energy (kilocalories normalized by body mass) consumed per kilometer and the Cost running above standing (Cr_net_). As expected for the last section of the race, no differences were observed in running economy between male and female runners, independently of the method used (Figure 3D,F, and Table 3). Females seemed to have better running economy than males in the last race section (from 40 km to the finish line), which is denoted by: (i) females consumed less energy per kilometer than males (0.822 ± 0.187 kcal·kg^−1^·km^−1^ versus 0.977 ± 0.210 kcal·kg^−1^·km^−1^; *p*-value = 0.017; Cohen’s d = 0.53), and (ii) females presented a lower Cr_net_ than males (3248 ± 0.794 j·kg^−1^·m^−1^ versus 3860 ± 0.875 j·kg^−1^·m^−1^; *p*-value = 0.022; Cohen’s *d* = 0.51). Maximum Gap values were observed in the last race section for both variables (−18.89% for kcal·kg^−1^·km^−1^; and −18.85% for Cr_net_). In this race section, the negative values of Gap denoted that males consumed more energy per distance (and therefore presented a lower running economy) than females.

## 4. Discussion

This observational study aimed at increasing our understanding on how females and males achieve their marathon goal. In this regard, we focused on analyzing the evolution of several accelerometry-estimated parameters (energy consumption, running intensity distribution, running economy, oxygen uptake), as well as pace-related variables (running speed, average change in speed), throughout a marathon race taking into account runner’s sex. For this purpose, we directly monitored female and male recreational runners throughout the entire marathon distance by using accelerometer-based devices. Moreover, we also collected split times of each runner (provided by the organizers of the Valencia Marathon).

Similar to previous studies [4,53], the average running speed was significantly higher in males compared to females. In this study, the difference rate in running speed between males and females was 11.24%. This percentage matched with values obtained in previous studies, which ranged from 8% to 14% [4,5,6,8,10,17]. However, this percentage of sex difference seems to be lower in elite compared to amateur marathoners. In fact, the female marathon world record (2:14:09, Brigid Kosgei, Chicago 2019) is only 9.32% slower than the male marathon world record (2:01:39, Eliud Kipchoge, Berlin 2018).

Sex variability in marathon performance may be explained by the well-known differences in several physiological traits [4,53,54,55]. Descriptive analyses showed that males presented higher maximum oxygen uptake, body mass index, muscle strength, and lower percentage of body fat than females. Muscle strength has been shown to determine the runner’s ability of displacement and thus running speed. In fact, a lower percentage of sex difference has been observed for swimming speed (6–7%), a physical activity in which the muscle strength component is considerably less crucial for succeeding compared to running [56,57].

However, according to the split times collected of each runner, males were more likely to slow their pace in the last part of the marathon race than females. Males started the marathon running at a 15.53% faster speed than females in the first race section (from start line to 5 km), and this difference rate was decreasing throughout the marathon. In fact, females run at 4.5% faster speed than males in the last marathon section (from 40 km to finish line). Additionally, by analyzing the ACS [9], we were able to confirm that females raced at a more constant pace from the start line to the 40 km as compared to males. This pace strategy may allow females to significantly increase their running speed in the last section of the race, while males were more likely to “hit the wall” in the last kilometers.

At this point, we would like to highlight the notably difference in the running speed maintained by males and females in the first section of the race (Gap of −15.53%). This difference may be attributed to the fact that, in races with more than 20,000 participants, runners cannot run the first kilometers freely without difficulty due to the large number of participants and the limited space. This notable sex difference may thus indicate that males started the race being more ambitious, while females adopted a more cautious attitude [54,55,58]. This observation was not previously seen by Nikolaidis and cols (2019) [9], probably because they split the race distance into 10 km sectors and not in 5 km sectors as we did in this work. Having shorter sections allowed us to observe changes in running pace and intensity in greater detail.

To further explore sex differences in marathon performance, and taking into account that there is a lack of gold standard for measuring energy consumption in free-living conditions (as a marathon race) [40], we used accelerometer-based devises for estimating the distribution of physical effort throughout the marathon according to runner’s sex [39]. Specifically, we estimated the time running at each of the six-relative intensity levels (sedentary, light, moderate, vigorous, very vigorous and extremely vigorous) in each one of the nine race sections and in the entire marathon. The analysis of physical effort distribution denoted that females tended to race at a lower intensity level than males (females significantly ran more percentage of time at very vigorous intensity than males, who mainly ran at extremely vigorous intensity).

In addition, accelerometry-based data allowed us to estimate the energy consumed and the %VO_2max_ sustained per each runner, and afterwards his/her running economy. According to our results, females reported a better efficiency of movement than males in the last section of the marathon (from 40 km to the finish line). That is, a superior energy was demanded by males for running at a given speed the last 2.195 km of the marathon. This may be a consequence of the high physical effort sustained by males in the first part of the marathon, pointing out the importance of controlling physical effort distribution in a marathon race to avoid “hitting the wall”. Running at high intensities has been shown to accelerate glycolytic depletion [55], which may contribute to the decrease of running pace observed in males in the last part of the marathon. Females, however, may use fats as principal energy source maintaining their glycogen stores in muscles thanks to running at less demanding intensities. As stated in lab-based conditions [13], females may present lower respiratory exchange ratio (RER) compared to males, indicating in turn that fat may be the principal fuel source used by females. Future work may be focused on validating accelerometry for RER estimations.

Two limitations are noteworthy in the present study. Firstly, we are aware about the low number of females included in our population (15.91%). However, this percentage is even higher than the rate of females, aged 30 to 45 years, finishing the Valencia Marathon in 2016 (13.16%). Higher effort should be done in future studies for increasing the number of females collected. The second limitation is that values of accelerometer-based parameters analyzed in this study were merely estimations. No gold standard method is available yet to perform a direct measurement of VO_2_ consumed by a runner in free-living conditions. We may assume a plausible maximum error of 10% in our estimations.

In summary, thanks to the accelerometry-based and pace-based data collected, this study reveals how female and male middle-aged amateur marathoners face a marathon in terms of pacing, running strategy, running intensity distribution, energy consumption and running economy. The use of accelerometer devices for monitoring runners allowed us to perform an individualized assessment in the context of free-living movement. In general, females showed a good control of physical effort throughout the marathon, while the running intensity distribution and pacing of males were not so well-balanced. Subsequently, an increased decay of running pace in the last part of the marathon was observed for males. Results may reflect well-known sex differences in physiology (i.e., muscle strength, fat metabolism, VO_2max_), and in running strategy approach (i.e., females run at a more conservative intensity level in the first part of the marathon compared to males).

## 5. Conclusions

Compared to males, females maintained a more stable pace and ran at less demanding running intensities throughout the marathon, limiting the decay of running pace in the last part of the race. Together with previous studies, the results obtained after analyzing a huge number of variables suggest that the steady pacing of females may be because of the following reasons:

- Females may manage energy during the race more efficiently than males [7,55].
- Females may make better decisions in terms of pacing strategy than males [6,48,54].
- Females typically use more fat than carbohydrates during endurance exercise compared to males [14,59,60].
- Females tend to preserve muscle strength and have less neuromuscular fatigue than males at the end of the marathon [61].

## Figures and Tables

**Table 1:** Values established for delineating the six-relative intensity levels of physical activity according to runner’s sex.

		Reference Values Established for Each Intensity Level in Males by Hernando et al. (2018)		Values used for Energy Consumption Estimation		
Sex	Relative-Intensity Levels of Physical Activity #	VO_2_ (mL·kg^−1^·min^−1^)	METs *	%VO_2max_ (mL·kg^−1^·min^−1^)	VO_2_ (mL·kg^−1^·min^−1^)	METs *
**Males**	Sedentary X < 10%	VO_2_ < 5.57	METs < 1.59	8.1	4.5	1.29
	Light 10% ≤ X <25%	5.57 ≤ VO_2_ <13.94	1.59 ≤ METs < 3.97	17.5	9.75	2.79
	Moderate 25% ≤ X < 45%	13.94 ≤ VO_2_ < 25.08	3.97 ≤ METs < 7.15	35.0	19.51	5.57
	Vigorous 45% ≤ X < 65%	25.08 ≤ VO_2_ < 36.23	7.15 ≤ METs < 10.33	55.0	30.66	8.76
	Very Vigorous 65% ≤ X < 85%	36.23 ≤ VO_2_ < 47.38	10.33 ≤ METs < 13.54	75.0	41.81	11.94
	Extremely Vigorous X ≥ 85%	VO_2_ ≥ 47.38	METs ≥ 13.54	92.5	51.56	14.73
**Females**	Sedentary X < 10%	VO_2_ < 4.82	METs < 1.38	8.1	3.91	1.12
	Light 10% ≤ X <25%	4.82 ≤ VO_2_ <12.07	1.38 ≤ METs < 3.45	17.5	8.44	2.41
	Moderate 25% ≤ X < 45%	12.07 ≤ VO_2_ < 21.72	3.45 ≤ METs < 6.21	35.0	16.89	4.83
	Vigorous 45% ≤ X < 65%	21.72 ≤ VO_2_ < 31.38	6.21 ≤ METs < 8.97	55.0	26.55	7.59
	Very Vigorous 65% ≤ X < 85%	31.38 ≤ VO_2_ < 41.03	8.97 ≤ METs < 11.72	75.0	36.20	10.34
	Extremely Vigorous X ≥ 85%	VO_2_ ≥ 41.03	METs ≥ 11.72	92.5	44.65	12.76

Abbreviations: N, number of individuals; VO_2max_, maximum oxygen consumption; VO_2_, oxygen consumption; MET, metabolic equivalent task. Each minute of the cardiopulmonary test was classified into one of the six intensity categories of physical activity relative to an individual’s level of cardiorespiratory (VO_2max_). # X denotes the percentage of an individual’s aerobic capacity (VO_2max_) used to classify each one of the six relative-intensity categories. * 1 MET = 3.5 mLO_2_·kg^−1^·min^−1^. 1 MET = 1 Kcal·h^−1^.

**Table 2:** Population description.

Variables		Males (N = 74)	Females (N = 14)	*p*-Value
Physiological characteristics *	age	38.58 ± 3.70	39.21 ± 3.14	0.61
	BMI	23.15 ± 1.46	21.65 ± 1.93	**0.001**
	% body fat	13.76 ± 3.68	19.94 ± 4.26	**2.03 × 10^−5^**
	VO_2max_ (mL·kg^−1^·min^−1^)	55.55 ± 5.25	48.39 ± 3.60	**6.59 × 10^−6^**
	maximum METs	15.87 ± 1.50	13.83 ± 1.03	**6.77 × 10^−6^**
Training indicators *	years of running	6.42 ± 2.89	6.43 ± 2.17	0.99
	sessions per week	4.97 ± 0.83	4.50 ± 0.76	**0.04**
	kilometers per week	64.32 ± 13.16	58.93 ± 11.96	0.14
	hours per week	7.54 ± 2.57	6.46 ± 1.82	0.16
History as marathoner *	marathons finished	3.62 ± 3.11	2.00 ± 2.15	**0.03**
	marathon per year	1.12 ± 0.64	1.00 ± 0.55	0.58
Work intensity #	high intensity	9.46%	0.00%	0.44
	medium intensity	31.08%	28.57%	
	low intensity	59.46%	71.43%	
Levels of study #	school graduate	4.11%	7.14%	0.72
	high school graduate	19.18%	7.14%	
	professional certificate	6.85%	7.14%	
	undergraduate degree	69.86%	78.57%	

Abbreviations: N, number of samples; BMI, body mass index; SD, standard deviation. * Values are presented as mean ± SD. #. Values are presented as percentage. Mann–Whitney U test was used for comparing quantitative variables among groups. Chi-square test was used for comparing categorical variables among groups. Bold denotes significant results.

**Table 3:** Comparison of the different variables collected over the whole marathon distance between males and females.

Variable	Males (N = 74)	Females (N = 14)	*p*-Value	Cohen’s *d*	Gap
Speed (m·min^−1^)	201.29 ± 17.84	180.96 ± 14.07	**1.74 × 10^−4^**	0.87	−11.24%
Energy consumed (kcal)	3274.07 ± 599.82	2423.01 ± 239.76	**9.32 × 10^−7^**	1.23	−35.12%
Relative energy consumed per minute (kcal·kg^−1^·min^−1^)	0.21 ± 0.03	0.19 ± 0.02	**9.91 × 10^−4^**	0.75	−14.42%
Relative energy consumed per kilometer (kcal·kg^−1^·km^−1^)	1.07 ± 0.16	1.04 ± 0.11	0.34	0.21	−2.91%
Cost running net (Cr_net_)	4.22 ± 0.69	4.11 ± 0.49	0.38	0.19	−2.82%
Percentage of VO_2max_ (%)	80.76 ± 11.51	81.57 ± 7.59	0.68	0.09	1.00%
Basal Metabolic Rate (BMR)	12.87 ± 1.84	11.24 ± 1.06	**8.29 × 10^−4^**	0.76	−14.47%
Marathon time (minutes)	211.28 ± 19.16	234.50 ± 18.46	**1.74 × 10^−4^**	0.87	9.90%
Squat jump at the start line (cm)	27.24 ± 4.29	23.84 ± 3.82	**0.007**	0.60	−14.26%
Squat jump at the finish line (cm)	21.89 ± 6.19	20.53 ± 6.72	0.30	0.22	−6.62%
Average change in speed (%)	5.39 ± 2.62	6.29 ± 2.58	0.15	0.31	14%
% of time at sedentary level	0.01 ± 0.12	0.00 ± 0.00	0.66	0.02	NA
% of time at light level	0.09 ± 0.5	0.07 ± 0.27	0.81	0.02	−29%
% of time at moderate level	3.61 ± 7.40	1.07 ± 1.59	0.11	0.33	−237%
% of time at vigorous level	11.58 ± 19.58	4.79 ± 8,11	0.10	0.35	−142%
% of time at very vigorous level	30.82 ± 29.33	50.50 ± 30.29	**0.02**	0.52	39%
% of time at extremely vigorous level	53.88 ± 39.29	43.64 ± 34.86	0.27	0.24	−23%

Abbreviations: N, number of samples; SD, standard deviation; NA, not available; Gap, percentage of sex differences. Values are presented as mean ± SD. Mann–Whitney U test was used for comparing quantitative variables among groups. Cohen’s *d* was calculated for inferring the effect size of a variable. Bold denotes significant results.

## Data Availability

All data generated or analyzed during this study are included in this published article (and its Supplementary Materials).

## Supplementary Materials

The following are available online at https://www.mdpi.com/1660-4601/17/17/6196/s1. Table S1: Evaluation of running intensity distribution and estimation of calories consumed by male runners based on accelerometry data. Table S2: Evaluation of running intensity distribution and estimation of calories consumed by male runners based on accelerometry data. File S1: Raw data of the study.
