# Supplementary material for: Using Accelerometry for Evaluating Energy Consumption and Running Intensity Distribution Throughout a Marathon According to Sex
Source: Int J Environ Res Public Health. 2020 Aug 26;17(17):6196. doi: 10.3390/ijerph17176196 (PMC7503696; doi:10.3390/ijerph17176196)
Supplement: Supplementary file 1 [file ijerph-17-06196-s001.zip › SupplemetaryMaterials/SupplementaryMaterials-Tables.docx]

Supplementary Materials for

Using accelerometry for evaluating energy consumption and running intensity distribution throughout a marathon according to sex

**Carlos Hernando^1,2^*, Carla Hernando^3^, Ignacio Martinez-Navarro^4,5^, Eladio Collado-Boira^6^, Nayara Panizo^6^, Barbara Hernando^7^**

^1^ Sport Service, Jaume I University, Castellon (Spain)

^2^ Department of Education and Specific Didactics, Jaume I University, Castellon (Spain)

^3^ Department of Mathematics, Carlos III University of Madrid (Spain)

^4^ Department of Physical Education and Sport, University of Valencia (Spain)

^5^ Sports Health Unit, Vithas-Nisa 9 de Octubre Hospital (Spain)

^6^ Faculty of Health Sciences, Jaume I University, Castellon (Spain)

^7^ Department of Medicine, Jaume I University, Castellon (Spain)

**This PDF file includes:**

Table S1. Evaluation of running intensity distribution and estimation of calories consumed by male runners based on accelerometry data

Table S2. Evaluation of running intensity distribution and estimation of calories consumed by male runners based on accelerometry data

**Other Supplementary Materials for this manuscript includes the following:**

File S1. Raw data of the study (demographics, marathon split times, accelerometry output data)

**Table S1.** Evaluation of running intensity distribution and estimation of calories consumed by male runners based on accelerometry data

| **Race section** | **Time spend at each relative-intensity level (minutes)** | | | | | | | **Energy consumed according to the time spend at each relative-intensity level (kcal)** | | | | | | |
| --- | --- | --- | --- | --- | --- | --- | --- | --- | --- | --- | --- | --- | --- | --- |
|  | **S** | **L** | **M** | **V** | **VV** | **EV** | **Total** | **S** | **L** | **M** | **V** | **VV** | **EV** | **Total** |
| **0-5km** | 0.01  ±0.12 | 0.00  ±0.00 | 0.46  ±1.23 | 2.88  ±5.93 | 9.03  ±9.39 | 14.05  ±11.12 | 26.43  ±2.81 | 0.02  ±0.17 | 0.00  ±0.00 | 3.08  ±7.93 | 31.22  ±65.56 | 131.81  ±140.18 | 249.22  ±199.97 | 415.35  ±74.47 |
| **5-10km** | 0.00  ±0.00 | 0.00  ±0.00 | 0.70  ±2.07 | 3.07  ±6.22 | 7.85  ±8.47 | 12.46  ±10.11 | 24.08  ±1.92 | 0.00  ±0.00 | 0.00  ±0.00 | 4.81  ±13.88 | 32.72  ±67.93 | 114.22  ±125.90 | 221.89  ±181.62 | 373.63  ±72.14 |
| **10-15km** | 0.00  ±0.00 | 0.00  ±0.00 | 0.92  ±2.26 | 3.26  ±6.23 | 7.66  ±8.17 | 12.42  ±10.10 | 24.26  ±2.03 | 0.00  ±0.00 | 0.00  ±0.00 | 6.33  ±15.53 | 34.88  ±67.54 | 111.54  ±121.73 | 220.72  ±181.41 | 373.47  ±71.75 |
| **15-HM** | 0.00  ±0.00 | 0.00  ±0.00 | 1.31  ±3.46 | 4.23  ±7.14 | 8.77  ±9.31 | 15.14  ±12.36 | 29.45  ±2.55 | 0.00  ±0.00 | 0.00  ±0.00 | 9.09  ±23.63 | 44.77  ±76.17 | 127.10  ±135.07 | 270.04  ±222.68 | 451.00  ±90.00 |
| **HM-25km** | 0.00  ±0.00 | 0.00  ±0.00 | 0.32  ±1.60 | 2.20  ±5.09 | 5.54  ±6.96 | 11.09  ±8.44 | 19.16  ±1.54 | 0.00  ±0.00 | 0.00  ±0.00 | 2.08  ±10.15 | 23.27  ±55.06 | 80.86  ±103.79 | 198.16  ±153.12 | 304.36  ±61.60 |
| **25-30km** | 0.00  ±0.00 | 0.01  ±0.12 | 0.82  ±2.30 | 3.35  ±5.56 | 7.51  ±7.64 | 13.31  ±10.12 | 25.01  ±2.27 | 0.00  ±0.00 | 0.04  ±0.37 | 5.52  ±15.25 | 35.56  ±59.93 | 108.75  ±110.71 | 238.18  ±182.88 | 388.06  ±75.53 |
| **30-35km** | 0.00  ±0.00 | 0.07  ±0.42 | 1.46  ±5.01 | 2.89  ±5.45 | 7.68  ±8.30 | 14.04  ±10.95 | 26.14  ±3.40 | 0.00  ±0.00 | 0.25  ±1.56 | 10.13  ±34.99 | 30.57  ±59.03 | 110.50  ±119.84 | 251.54  ±198.56 | 402.98  ±82.36 |
| **35-40km** | 0.00  ±0.00 | 0.11  ±0.63 | 1.96  ±5.60 | 2.59  ±4.49 | 8.15  ±8.33 | 14.00  ±10.33 | 26.81  ±3.98 | 0.00  ±0.00 | 0.40  ±2.46 | 13.64  ±39.05 | 27.19  ±47.09 | 117.47  ±121.83 | 250.61  ±186.84 | 409.30  ±75.14 |
| **40-M** | 0.03  ±0.23 | 0.03  ±0.16 | 0.53  ±1.77 | 0.92  ±1.86 | 2.45  ±3.07 | 6.00  ±4.14 | 9.95  ±1.76 | 0.04  ±0.33 | 0.09  ±0.53 | 3.63  ±12.44 | 9.62  ±19.45 | 35.36  ±44.82 | 107.19  ±74.69 | 155.93  ±36.55 |
| **Marathon** | 0.04  ±0.35 | 0.22  ±1.00 | 8.49  ±18.38 | 25.39  ±43.48 | 64.64  ±60.41 | 112.51  ±82.02 | 211.28  ±19.16 | 0.06  ±0.50 | 0.78  ±3.86 | 58.31  ±125.75 | 269.79  ±471.40 | 937.60  ±888.65 | 2007.54  ±1474.36 | 3274.07  ±599.82 |
| Abbreviations: S, Sedentary; L, Light; M, Moderate; V, Vigorous; VV, Very Vigorous; EV, Extremely Vigorous; HM, Half marathon; M, marathon; SD, standard deviation.  Values are presented as mean ± SD  Marathon time of 211.28 ± 19.55 min, body mass of 74.07 ± 6.94 kg, and BMI of 23.15 ± 1.46 kg·m-2 (N=74) | | | | | | | | | | | | | | |

**Table S2.** Evaluation of running intensity distribution and estimation of calories consumed by male runners based on accelerometry data

| **Race section** | **Time spend at each relative-intensity level (minutes)** | | | | | | | **Energy consumed according to the time spend at each relative-intensity level (kcal)** | | | | | | |
| --- | --- | --- | --- | --- | --- | --- | --- | --- | --- | --- | --- | --- | --- | --- |
|  | **S** | **L** | **M** | **V** | **VV** | **EV** | **Total** | **S** | **L** | **M** | **V** | **VV** | **EV** | **Total** |
| **0-5km** | 0.00  ±0.00 | 0.00  ±0.00 | 0.07  ±0.27 | 3.00  ±7.07 | 15.29  ±12.76 | 12.29  ±13.95 | 30.64  ±3.85 | 0.00  ±0.00 | 0.00  ±0.00 | 0.33  ±1.23 | 22.58  ±54.87 | 149.81  ±126.15 | 138.49  ±155.14 | 311.21  ±45.19 |
| **5-10km** | 0.00  ±0.00 | 0.00  ±0.00 | 0.29  ±0.61 | 2.71  ±4.76 | 13.71  ±9.56 | 10.50  ±10.97 | 27.21  ±1.93 | 0.00  ±0.00 | 0.00  ±0.00 | 1.16  ±2.40 | 19.56  ±34.43 | 135.73  ±98.42 | 118.60  ±122.39 | 275.05  ±34.93 |
| **10-15km** | 0.00  ±0.00 | 0.00  ±0.00 | 0.00  ±0.00 | 0.79  ±2.42 | 16.50  ±10.65 | 10.00  ±10.86 | 27.29  ±2.13 | 0.00  ±0.00 | 0.00  ±0.00 | 0.00  ±0.00 | 6.05  ±18.96 | 161.17  ±107.34 | 114.06  ±123.81 | 281.29  ±31.57 |
| **15-HM** | 0.00  ±0.00 | 0.14  ±0.54 | 0.00  ±0.00 | 2.29  ±4.43 | 16.57  ±12.65 | 14.00  ±13.50 | 33.00  ±2.69 | 0.00  ±0.00 | 0.31  ±1.15 | 0.00  ±0.00 | 16.90  ±33.51 | 165.25  ±129.86 | 157.08  ±148.33 | 339.52  ±34.79 |
| **HM-25km** | 0.00  ±0.00 | 0.07  ±0.27 | 0.07  ±0.27 | 0.36  ±0.63 | 11.00  ±9.78 | 9.93  ±9.50 | 21.43  ±1.74 | 0.00  ±0.00 | 0.17  ±0.65 | 0.31  ±1.15 | 2.62  ±4.80 | 110.71  ±100.31 | 111.14  ±105.66 | 224.96  ±18.26 |
| **25-30km** | 0.00  ±0.00 | 0.00  ±0.00 | 0.29  ±0.83 | 0.57  ±0.94 | 16.07  ±9.33 | 11.07  ±9.06 | 28.00  ±2.22 | 0.00  ±0.00 | 0.00  ±0.00 | 1.27  ±3.60 | 4.14  ±7.09 | 158.18  ±96.92 | 125.94  ±100.43 | 289.54  ±23.04 |
| **30-35km** | 0.00  ±0.00 | 0.00  ±0.00 | 0.43  ±0.94 | 0.57  ±1.02 | 14.14  ±10.88 | 13.36  ±10.83 | 28.50  ±3.11 | 0.00  ±0.00 | 0.00  ±0.00 | 1.96  ±4.40 | 4.28  ±7.71 | 140.42  ±11.80 | 151.95  ±121.09 | 298.61  ±31.49 |
| **35-40km** | 0.00  ±0.00 | 0.00  ±0.00 | 0.57  ±0.94 | 0.93  ±1.49 | 13.14  ±9.55 | 14.21  ±9.90 | 28.86  ±2.88 | 0.00  ±0.00 | 0.00  ±0.00 | 2.67  ±4.47 | 6.71  ±10.82 | 128.73  ±96.61 | 164.28  ±113.57 | 302.39  ±30.80 |
| **40-M** | 0.00  ±0.00 | 0.07  ±0.27 | 0.71  ±2.67 | 0.21  ±0.58 | 2.64  ±2.90 | 5.93  ±3.79 | 9.57  ±1.83 | 0.00  ±0.00 | 0.18  ±0.67 | 3.57  ±13.34 | 1.63  ±4.47 | 25.71  ±28.98 | 69.37  ±45.42 | 100.45  ±24.18 |
| **Marathon** | 0.00  ±0.00 | 0.29  ±0.61 | 2.43  ±3.67 | 11.43  ±19.67 | 119.07  ±73.16 | 101.29  ±81.69 | 234.50  ±18.46 | 0.00  ±0.00 | 0.66  ±1.38 | 11.26  ±17.66 | 84.47  ±151.88 | 1175.72  ±751.08 | 1150.90  ±803.61 | 2423.01  ±239.76 |
| Abbreviations: S, Sedentary; L, Light; M, Moderate; V, Vigorous; VV, Very Vigorous; EV, Extremely Vigorous; HM, Half marathon; M, marathon; SD, standard deviation.  Values are presented as mean ± SD  Marathon time of 234.50 ± 18.46 min, body mass of 56.80 ± 4.27 kg, and BMI of 21.65 ± 1.93 kg·m-2 (N=14) | | | | | | | | | | | | | | |
